# Microelectrode Array Recordings from the Ventral Roots in Chronically Implanted Cats

**DOI:** 10.3389/fneur.2014.00104

**Published:** 2014-07-07

**Authors:** Shubham Debnath, Matthew J. Bauman, Lee E. Fisher, Douglas J. Weber, Robert A. Gaunt

**Affiliations:** ^1^Department of Bioengineering, University of Pittsburgh, Pittsburgh, PA, USA; ^2^Department of Physical Medicine and Rehabilitation, University of Pittsburgh, Pittsburgh, PA, USA

**Keywords:** ventral root, motor neuron, single unit recording, impedance, peripheral nerve interface

## Abstract

The ventral spinal roots contain the axons of spinal motoneurons and provide the only location in the peripheral nervous system where recorded neural activity can be assured to be motor rather than sensory. This study demonstrates recordings of single unit activity from these ventral root axons using floating microelectrode arrays (FMAs). Ventral root recordings were characterized by examining single unit yield and signal-to-noise ratios (SNR) with 32-channel FMAs implanted chronically in the L6 and L7 spinal roots of nine cats. Single unit recordings were performed for implant periods of up to 12 weeks. Motor units were identified based on active discharge during locomotion and inactivity under anesthesia. Motor unit yield and SNR were calculated for each electrode, and results were grouped by electrode site size, which were varied systematically between 25 and 160 μm to determine effects on signal quality. The unit yields and SNR did not differ significantly across this wide range of electrode sizes. Both SNR and yield decayed over time, but electrodes were able to record spikes with SNR >2 up to 12 weeks post-implant. These results demonstrate that it is feasible to record single unit activity from multiple isolated motor units with penetrating microelectrode arrays implanted chronically in the ventral spinal roots. This approach could be useful for creating a spinal nerve interface for advanced neural prostheses, and results of this study will be used to improve design of microelectrodes for chronic neural recording in the ventral spinal roots.

## Introduction

In 2005, nearly two million people in the United States were living with the loss of a limb, and it is projected that this number will double by 2050 (1) due to amputations following vascular disease, trauma, and cancer. In order to restore function to these individuals, neural interface technologies are being developed to enable intuitive control of robotic prostheses (2–7). These neural interfaces extract control signals from the nervous system by decoding motor intent from the signals recorded in the brain (3, 4, 8, 9), peripheral nerves (2), or muscles (5, 10).

The spinal nerves, and specifically the ventral roots, provide a potentially compelling target for a neural interface to extract motor control signals from the nervous system. Because of the unique organization of the spinal roots, a ventral root interface would have access to a large numbers of motor nerve fibers, which are packed densely in the ventral roots and are physically isolated from sensory fibers located in the adjacent dorsal roots. Further, neural activity in the motor axons of the ventral root leads directly to muscle contraction and could therefore provide a source for motor control signals that are linked directly to normal musculoskeletal action, including force production and, ultimately, limb motion. Additionally, the vertebral bones surrounding the spinal nerves provide mechanical protection for the implanted electrodes and a degree of electrical isolation to reduce EMG interference. There are also well-established minimally invasive spine surgery procedures (11) that could potentially be adapted for implantation of electrodes in the spinal roots.

In the 1980s, Hoffer, Loeb, and colleagues conducted a set of experiments, where they recorded simultaneously from up to 12 penetrating “hatpin” microelectrodes implanted chronically in the L5 ventral roots of cats (12–15). These studies were performed to study motor unit recruitment physiology in locomoting cats and demonstrated initial feasibility for chronic ventral root recordings. While allowing the cats to move freely, individual units could be recorded for a whole day or longer, allowing recording to occur during a range of activities and over long periods of time. The studies found that the modulation of firing frequency closely resembled modulation in EMG amplitude recorded in individual leg muscles. Motor unit recordings were made in ventral root axons over several months, but the chronic stability of these recordings was never characterized.

The primary aim of this study was to characterize the motor unit recording performance of high-density microelectrode arrays implanted chronically in the ventral roots of awake behaving cats. Different electrode tip exposure lengths were tested with the hypothesis that larger electrode tips would be more likely to record neural activity than smaller electrode tips. Signal-to-noise ratios (SNR), unit yield, and electrical impedance were measured for the duration of the implants. The primary outcome of this study was that high SNR motor unit signals were recorded in nearly all implants during treadmill locomotion, although there was significant variability between implants. Electrode tip exposure lengths (site sizes) had minimal impact on the ability to record single unit activity and electrode array positioning was the most important factor in achieving robust single unit neural recordings.

## Materials and Methods

The objective of this study was to evaluate the neural recording capability of floating microelectrode arrays (FMAs) implanted chronically in the ventral roots of cats as assessed by motor unit yield and SNR over time. All procedures were approved by the University of Pittsburgh Institutional Animal Care and Use Committee and the US Army Medical Department Animal Care and Use Review Office.

### Electrode design and pre-operative testing

The FMA (Microprobes for Life Science, Gaithersburg, MD, USA) is a direct descendant of the “hatpin” electrode technology used by Hoffer, Loeb, and colleagues in their ventral root studies. The FMA is comprised of conventional “stiff” platinum–iridium microelectrodes mounted into a ceramic substrate with a flexible set of gold lead wires, allowing the array to “float” within the neural tissue. Importantly for this study, the FMA allows user-defined electrode lengths, facilitating a dorsal approach to ventral root electrode implantation, inserting the electrodes through the dorsal root ganglia (DRG) into the ventral root. The recording electrode lengths varied from 2.3 to 3.5 mm, which allowed them fully penetrate the DRG and span the depth of the ventral root. The L7 arrays (2.8–3.5 mm) were longer than L6 arrays (2.3–3.0 mm). Each array included two reference and ground electrodes at the corners, which were 3.7 mm long.

**Figure 1:**
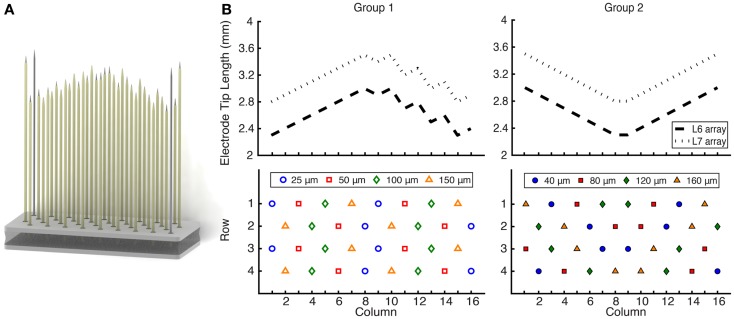
**(A)** Rendering of a 32-channel FMA that was implanted in the L6 and L7 ventral roots of nine cats. **(B)** Electrode length profile and site size layout. The electrode lengths were customized to reach the ventral root through the DRG. L7 arrays (2.8–3.5 mm) were longer than L6 arrays (2.3–3.0 mm), and the profile was updated for group 2 implants. Electrode recording site sizes varied from 25 to 150 μm for group 1 and 40 to 160 μm for group 2, for which the layout was also changed. Each array also included two ground and two reference electrodes at the corners.

It was hypothesized that the geometry of the exposed tip length (or site size) would have an impact on the quality and number of neurons recorded. For recordings from axons, these dimensions may be especially important since high SNR neural activity is most likely to be recorded near a node of Ranvier, where the current densities are highest. The 32 recording electrodes on each FMA (Figure 1) were spaced equidistantly at 400 μm and had a variety of site sizes, which were different for each of two groups. Group 1 electrode arrays had site sizes of 25, 50, 100, and 150 μm, while group 2 electrode arrays had site sizes of 40, 80, 120, and 160 μm. Reference electrodes had exposures of 500 μm, while ground electrodes were completely uninsulated.

Before implantation of the FMAs, each array was inspected through a microscope to check for bent or broken electrodes and to examine the integrity of the wire bundle. Additionally, the electrode connectivity was confirmed prior to implantation by measuring impedances of all electrodes before implantation. A multi-channel potentiostat (niPOD, NeuroNexus Technologies, Ann Arbor, MI, USA) was used to measure electrical impedances *in vitro* at 1 kHz with the electrode array in a normal saline solution (0.9% sodium chloride). To form a three-electrode system, a reference electrode (silver/silver chloride) was also placed in the solution with current supplied by a gold counter electrode.

### Surgery and electrode placement

Nine adult male cats (3–6 kg) were included in this study. Anesthesia was induced with ketamine (20 mg/kg, IM), followed by intubation and continuous administration of isoflurane (1–2.5%) for the duration of the implant surgery. Respiratory rate, expired CO_2_, O_2_ saturation, blood pressure, heart rate, and rectal temperature were monitored throughout the procedure and maintained within normal physiological ranges.

After revealing the spinal lamina by reflecting the paraspinal muscles overlying the L5–S1 vertebrae, a dorsal laminectomy was performed to expose the left L6 and L7 spinal roots. While direct access to the ventral root with flexible wire electrodes has been previously demonstrated without traversing the DRG (16), FMAs were too large to use this approach. Penetration of the DRG to reach the ventral root has also been reported (12) and leads to stable implants, and thus, in this study, the FMA was inserted through the DRG and into the ventral root. A custom vacuum holder attached to a micromanipulator was used to position the FMA over the DRG during visualization through a surgical microscope. A pneumatic-actuated inserter with 1.5 mm of travel (Blackrock Microsystems, Salt Lake City, UT, USA) was used to rapidly insert the FMAs through the epineurium by impacting the back end of the vacuum holder. High-speed insertion was required because the epineurium of the spinal roots cannot be penetrated using traditional slow, but continuous, insertion techniques used for cortical implantation of the FMAs. For chronic implantation, peeling back the epineurium was avoided. While it would have enabled slow insertion of the arrays, the process could have led to damage to the spinal roots themselves. After initial insertion, intraoperative electrophysiological recordings were performed to confirm the location in the ventral root. To successfully target the ventral root, the FMA must first travel through the DRG. Under surgical anesthesia, sensory afferents, but not motor efferents, within the spinal nerves remain active and responsive to hindlimb manipulation. To ensure that the electrode tips were located in the ventral root, the FMAs were advanced incrementally using the pneumatic inserter until only the shortest electrodes recorded sensory activity, or evoked neural activity was absent on all electrode channels.

With the FMAs inserted, the wire bundles were secured to the dura with 8–0 silk suture. A stainless steel wire (AS 636, Cooner Wire Company, Chatsworth, CA, USA) was attached to a bone screw in the iliac crest to act as an additional ground electrode, and a recording reference wire (AS 632, Cooner Wire Company, Chatsworth, CA, USA) was placed in the epidural space near the spinal cord. All connectors and external wires were passed through a percutaneous port and gathered into a custom housing unit. This protective plastic backpack was mounted on percutaneous posts anchored to a baseplate attached subcutaneously to the iliac crests and dorsal fascia. After surgery, animals were typically walking within 6–12 h and displayed essentially normal gain within 3–5 days.

### Neural signal recording

Before surgical implantation of the FMA, the cats were trained to walk on a treadmill at speeds ranging from 0.2 to 1.4 m/s. Cats were trained to walk continuously for 15 min, 5 days per week.

Neural signals from the microelectrode arrays were recorded with an OmniPlex D data acquisition system (Plexon Inc., Dallas, TX, USA) at 40 kHz and monitored constantly during all trials. Neural data were bandpass filtered between 300 Hz and 6 kHz. Awake trials consisted of three testing blocks. First, the cat would walk on the treadmill for up to 5 min at speeds ranging from 0.2 to 1.4 m/s (slower immediately after surgery but up to maximum speeds within a week). Second, the cat would stand quietly on all four legs. Third, the cat stood on its hindlimbs while leaning against a wall of the enclosure around the treadmill. The two standing conditions were used to provide recording of relatively static activation of the muscles, compared to phasic muscular activity that occurs during walking. High-speed walking and bipedal standing conditions were also used to generate high force activation of the hindlimb muscles in order to recruit higher threshold motor units that might not be activated during quiet standing. Awake trials were performed two to four times per week.

Anesthetized trials were completed one to three times per week and provided a method to classify recordings as either motor or sensory based on the knowledge that motor units are quiescent under anesthesia while sensory afferents remain modulated by limb movement. After completing all three awake blocks, the cat was lightly anesthetized with an injection of dexdomitor (40 μg/kg, IM). First, a baseline trial lasting 1 min was recorded without any stimulus or movement. Second, the implanted left leg was manipulated by alternately flexing and extending the entire leg at a moderate pace, pausing momentarily at each reversal point. The leg was continually maneuvered in this pattern for up to a minute while neural signals were recorded. Finally, neural activity was recorded while the leg was moved in a random pattern and manipulated in various motions, including flexing and extending different joints at different speeds and cycling rostrocaudally. Based on recordings during awake and anesthetized trials, units were classified as motor if they were active only during awake trials and sensory if they additionally responded during anesthetized movements.

### Electrode impedance measurements

While the cat was anesthetized, electrical impedances were recorded for each of the 32 electrodes on each array. Impedances were measured at a frequency of 1 kHz. The array’s reference and ground electrodes were employed as reference and ground points for *in vivo* impedance measurements. The niPOD (NeuroNexus Technologies, Ann Arbor, MI, USA) was used for impedance measurements for the first seven subjects, while other multi-channel potentiostat systems (Multi System, Metrohm Autolab, Utrecht, The Netherlands and CompactStat, Ivium Technologies, Eindhoven, The Netherlands) were used for trials in the last two subjects. All systems were compared with test electrodes *in vitro* and produced similar impedance results. Only functional electrodes with impedance <2 MΩ and >10 kΩ were included for all analyses. Electrodes with impedances above 2 MΩ were considered to be broken as a likely result of mechanical damage to the lead wire or electrode tip, while electrodes with impedances below 10 kΩ were believed to have failed due to delaminated insulation or other factors. This criterion allowed the inclusion of the maximum number of functional electrodes at each post-implant time point while ensuring that failed electrodes did not confound the data analysis. A more stringent impedance criterion was tested where the upper bound was varied by site size (0.5, 1, 1.5, 2 MΩ for largest to smallest electrodes), but no difference was seen in the results, so a 2 MΩ upper limit was used for all electrode sizes.

The impedance data were not normally distributed, as confirmed by Chi-square goodness-of-fit test for a normal distribution. Therefore, a Kruskal–Wallis analysis of variance (ANOVA) was performed to test effects of electrode site size and time on impedance measurements, followed by *post hoc* testing by the Mann–Whitney *U* test. Resulting *p*-values <0.05 were treated as significant.

### Neural signal processing and analysis

Individual action potentials were extracted from the data stream by detecting amplitude threshold crossings. The threshold was set to −3.5 times the standard deviation (σ) of the continuous data, and a spike event was stored each time this threshold was crossed. Each spike event consisted of a time stamp and an 800 μs snippet of voltage data before and after the threshold crossing.

All blocks (awake and anesthetized) for a single day were merged and sorted together using Offline Sorter (Plexon Inc., Dallas, TX, USA), as units typically remained on the same channel for all trials during 1 day. Cross channel artifacts were invalidated by removing spike events occurring on at least 25% of channels within a 75 μs window. Spike sorting was performed using the first three principal components of the snippet waveforms along with the voltage traces. Many channels contained activity from multiple single units, and these clusters were verified by hand sorting. Units that exhibited modulated activity during anesthetized test recordings were classified as sensory units and eliminated from further analysis.

The signal amplitude for each sorted unit was defined as the average extremum of all the individual action potential waveforms. Because the action potential is polyphasic, the extremum value could occur at a positive or negative voltage. The noise amplitude was set to 3σ of the filtered neural signal once all identified spikes were removed. Spiking activity was removed from the data signal prior to the noise amplitude estimation, as channels with highly active units could lead to overestimation of the noise. The SNR for a given unit was defined as the signal amplitude divided by the noise amplitude, which is an approach that has been used previously (17).

Single unit yield was quantified as the number of individual neural signals on each electrode that were classified as exhibiting motor-related activity. Units only counted toward yield if they had an SNR >1.2, as units with a lower SNR were typically poorly isolated. It should be noted that these poorly isolated often exhibited activity that was clearly modulated during motor tasks and could be useful for providing neural signals to control a prosthetic device. The SNR and single unit yield calculations for all testing blocks for all cats were aggregated by week, and median values are reported, unless otherwise specified.

A nearest-neighbor analysis was also conducted to examine the relationship between electrodes that successfully recorded single unit activity. With the dorsal implantation approach used in this study, the ability to record from motor axons requires electrodes to be precisely targeted within the ventral root, which is a significantly smaller target (~1 mm diameter) than the overlying DRG (~3 mm diameter) through which the electrode must pass. Well-positioned electrodes recording motor units were expected to be spatially clustered together on each array such that they were co-located within the ventral root. To assess the importance of electrode location on the ability to target and record from motor axons, the percentage of neighboring electrodes that recorded motor unit activity was computed for three groups of electrodes: those that recorded any single unit activity, those that specifically recorded motor unit activity, and those that did not record motor unit activity. An ANOVA was used to statistically compare these groups. Each electrode had up to six neighboring electrodes within 500 μm (see Figure 1) with electrodes in the center of the array having six nearest neighbors and electrodes on the edge or in the corners having from two to five nearest neighbors.

## Results

The ability to record action potentials from the axons of motor neurons in the ventral root was assessed by analyzing signal quality based on SNR and single unit yield over time. FMAs were implanted chronically in the left L6 and L7 ventral roots of nine cats. Neural recordings were performed approximately weekly for 4–12 weeks, and signal quality metrics were compared over a variety of electrode site sizes. Electrodes were excluded from analyses at any time point when the impedances were <10 kΩ or >2 MΩ.

### SNR and unit yield over time

**Figure 2:**
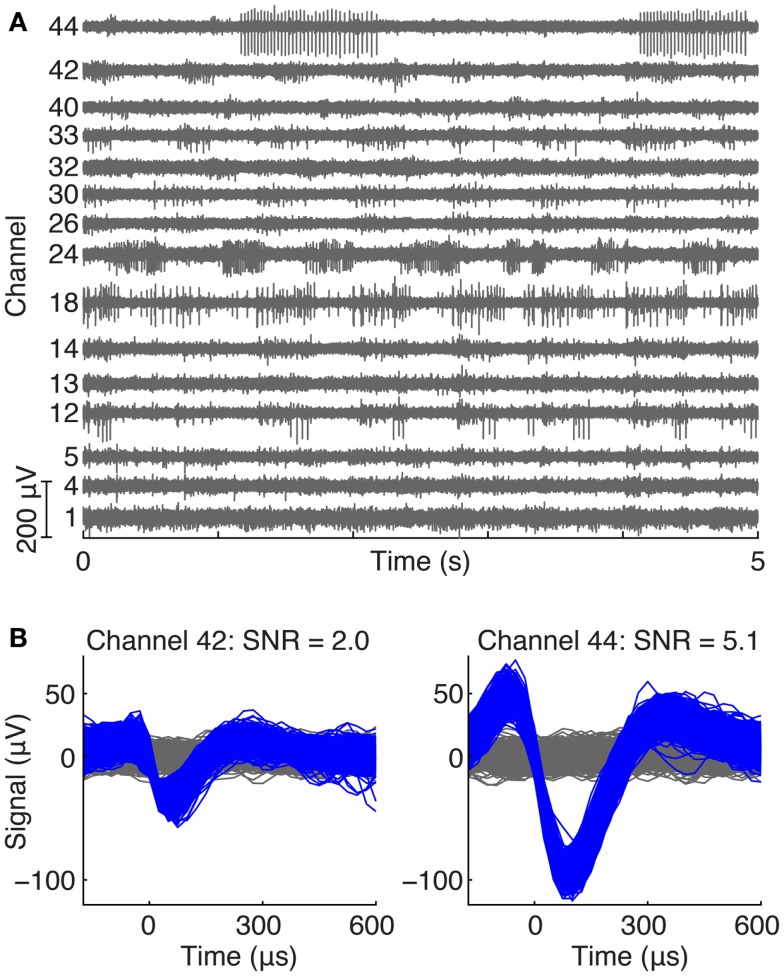
**(A)** Example of ventral root recordings during an awake trial. Phasically modulated spiking activity occurred on many channels during a treadmill locomotion trial. **(B)** Typical action potential waveforms recorded from motor axons in the ventral root with their respective SNRs.

Figure 2 shows an example of neural recordings during a treadmill locomotion trial. Figure 2A displays 5 s of raw voltage waveforms on 15 of 64 channels in cat V. All channels show neural spiking activity that was phasically modulated during the locomotor step cycle. Figure 2B shows the action potential waveforms extracted from two of the electrodes, along with their respective SNR values.

**Figure 3:**
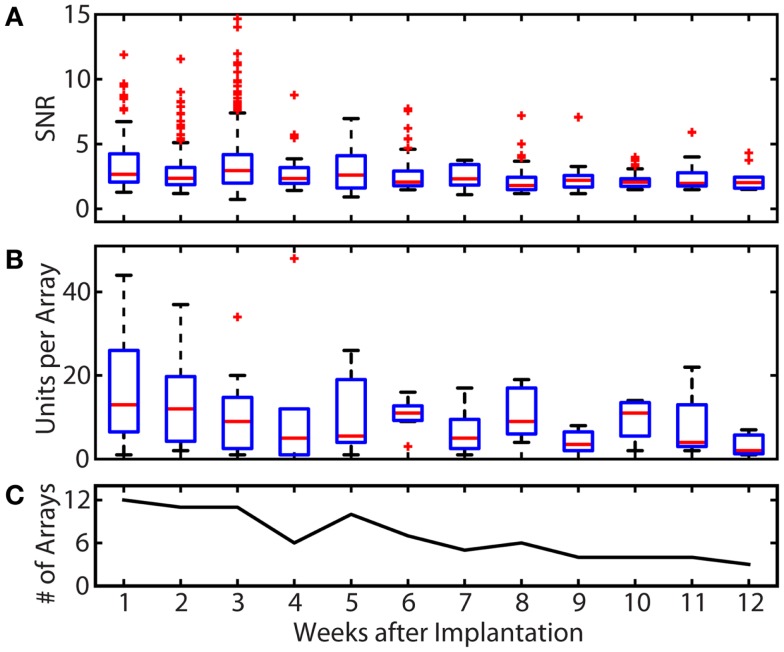
**Summary of recording quality and quantity for the duration of all implants**. **(A)** Summary of the SNR of all units over time. The median SNR (shown by the red line) remained at 2 or higher over the lifetime of all implants. Some single unit recordings with very high SNR values were observed in the first 4 weeks post-implantation. **(B)** Summary of motor unit yields over time. Single unit yield was calculated using units with an SNR >1.2. The mean number of units per array was 17.3 in week 1 and remained between 10.5 and 13.5 through week 6. The total single unit yield decayed over the implant periods, resulting in a mean of 3.3 units per array at week 12. **(C)** Number of arrays recorded from per week. Because the total implant time in each animal and the number of recording session per week varied, only available arrays were included in unit yield and SNR calculations. These variations explain the increases in yield between weeks 5 and 6 (and other weeks), which reflects the elimination of failed implants from the pool of arrays being evaluated. In the boxplots of **(A,B)**, the red line represents the median values, the upper and lower limits of the boxes represent the 75th and 25th percentiles, and the whiskers extend to cover approximately 99.3% of the data. Red “+” signs are data that fall outside of this range and are considered outliers.

Figure 3 summarizes the SNR and single unit yield for all motor units recorded from all cats throughout the study. Of 2726 total single units recorded in the 9 cats, 1277 (46.8%) were classified as motor units, having exhibited activity only during awake trials. Figure 3A shows the stability of the SNR for all motor units over time. The median SNR (shown by the red line) remained at 2 or higher over the lifetime of all implants, out to a maximum post-implant time of 12 weeks. In the first 4 weeks after implantation, very high SNR values ( >5) were frequently observed and are shown by the outliers denoted by red “+” signs.

The number of single units with an SNR >1.2 that were recorded on each array for each post-implant week is shown in Figure 3B. In the first post-implant week, there was a median of 13 units per array. Of the 12 recording weeks, the median number of units per array was >10 for 6 weeks. Only 1, 2, and 7 motor units per array remained on the three implanted arrays that lasted to the end of the 12-week study.

While the goal of the study was to track signal quality of the implanted electrodes over 3 months, implant lifetimes were highly variable from subject to subject. Table 1 shows the implant durations for all electrode arrays and the reasons for termination of each experiment. Figure 3C shows the total number of functioning electrode arrays tested at each time point after implantation and included in the analysis. Only two cats reached the end of the planned 3-month implantation period.

**Table 1:** **Summary of data collection intervals, initial yield description, and reasons for terminating experiments**.

	Group number	Implant duration (weeks after implantation)	Initial ventral root yield	Reason for termination
Cat W	1	12	Good	End of study
Cat V	1	8	Good	Slow signal degradation
Cat U	1	5	Moderate	Broken leads
Cat T	1	6	Good	Hardware/connector failure
Cat S	2	6	Moderate	Broken leads
Cat R	2	9	Good	Slow signal degradation
Cat Q	2	6	Poor	Immune reaction to surgery
Cat P	2	12	Moderate	End of study
Cat O	2	8	Poor	Slow signal degradation

### Effects of nearest-neighbor electrodes

The ability to successfully target the ventral root and the importance of the relative location of individual electrodes with respect to one another was determined using a nearest-neighbor analysis. Averaged across implanted arrays, 13% of an electrode’s neighbors recorded motor activity during awake treadmill walking trials. However, given that an electrode itself recorded motor activity, 35% of its neighboring electrodes recorded motor activity, which was a significantly higher percentage (*p* < 0.01). If an electrode did not record motor activity, the percentage of neighboring electrodes recording motor activity was only 10%, which was significantly less than the previous two groups (*p* < 0.01). These results suggest that if an electrode was successfully positioned in the ventral root and recorded motor activity, its neighbors also frequently recorded motor activity. This behavior was observed for 15 of the 18 implanted arrays.

### Effect of site size on SNR and unit yield

**Figure 4:**
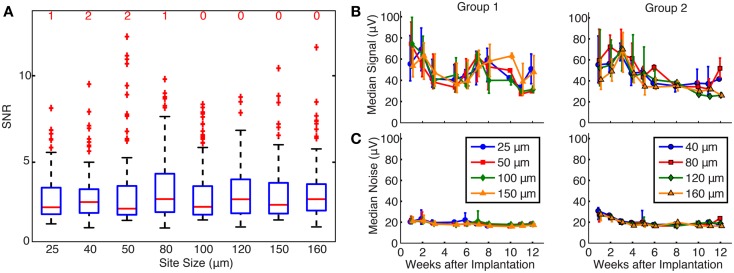
**(A)** Effects of site size on SNR. The red line represents the median values, the upper and lower limits of the boxes represent the 75th and 25th percentiles, and the whiskers extend to cover approximately 99.3% of the data. Red “+” signs are data that fall outside of this range and are considered outliers. All site sizes recorded units with median SNR between 2 and 4. The number of high SNR units (SNR >15) is shown by the red number above each box for each site size. By statistical testing, there was no effect of site size on SNR (*p* = 0.56). **(B)** Signal calculated over time for each site size. Median signal amplitude decreased over time for all site sizes, and site size was not a significant factor upon signal amplitude (*p* = 0.47 and 0.45 for groups 1 and 2, respectively). **(C)** Noise calculated over time for each site size. Median noise remained at approximately 20 μV for the duration of the implants, and site size did contribute to noise in group 1 electrodes (*p* < 0.01), but not group 2 electrodes (*p* = 0.17).

The effects of site size on SNR and unit yield are shown in Figure 4. Because SNR data were not normally distributed (Chi-square goodness-of-fit test), Kruskal–Wallis analysis was used to show that there was no significant effect of site size upon SNR (*p* = 0.56), and all site sizes recorded units with median SNR values between 2 and 4, as shown in Figure 4A. The number of units with an SNR >15 is shown by the red number above each box for each site size.

The effects of site size on signal and noise were also examined separately. The median signal amplitude (Figure 4B) decreased over time for all site sizes, while the median noise (Figure 4C) remained constant near 20 μV for the lifetime of the implants. Signal and noise data were not normally distributed (Chi-square goodness-of-fit test) and statistical testing using a Kruskal–Wallis analysis showed that site size was not a significant factor upon signal amplitude in both groups of electrodes (*p* = 0.47 and 0.45 for groups 1 and 2, respectively). Site size had a significant effect on the noise levels for group 1 (*p* < 0.01) but not for group 2 (*p* = 0.17) electrodes. *Post hoc* testing using the Mann–Whitney *U* test confirmed that noise levels were larger for smaller site sizes in group 1. Signal and noise measurements were also examined in a restricted time window (2–3 weeks post-implant) when signal quality was best and few electrode arrays had failed. Even in this time period, linear regressions between signal amplitude and site size were not significant for group 1 (*p* = 0.47) or group 2 (*p* = 0.69) electrodes. Linear regression between noise and site size was not significant for group 2 electrodes (*p* = 0.51) but was significant for group 1 electrodes (*p* = 0.04), mirroring the statistical analysis described above.

**Figure 5:**
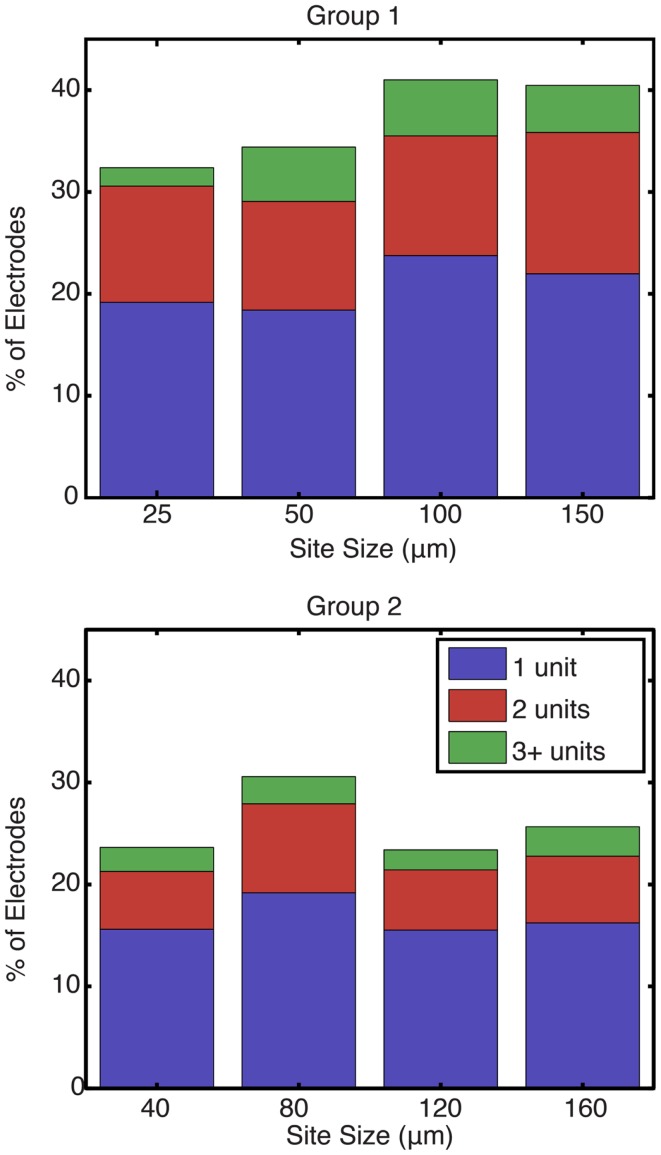
**Percentage of electrodes recording at least one unit by site size**. For each site size, each bar represents the total percentage of electrodes that recorded at least one unit, while the stacks represent the percentage of functional electrodes that were able to record one, two, or more than three units in blue, red, and green, respectively. Site size was not found to be a significant factor on the number of units recorded per electrode (*p* = 0.45).

Figure 5 shows the percentage of electrodes for each site size that were able to record one, two, or three or more single units. Each bar represents the total percentage of electrodes on an implanted array that recorded at least one unit. Statistical testing using an ANOVA showed that site size was not a significant factor on the number of units recorded per electrode (*p* = 0.45). An average of 37.1 and 25.8% of the electrodes in group 1 and 2 arrays, respectively, recorded at least one motor unit, while an average of 16.2 and 9.2% of the electrodes in group 1 and 2 arrays, respectively, recorded two or more motor units.

### Effect of site size on chronic electrode impedances

**Figure 6:**
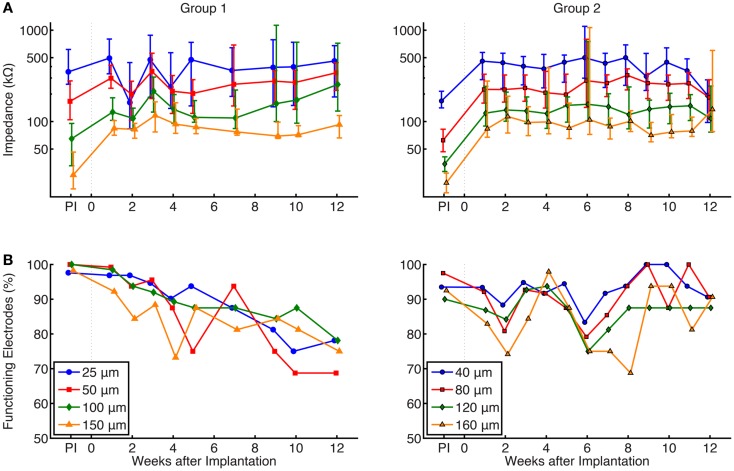
**(A)** Summary of chronic impedance measurements of all arrays. The line plots show the median impedance of the eight different electrode site sizes, with error bars representing the 75th and 25th percentiles of the data. The pre-implant (PI) impedance values, measured immediately before preparation for surgical implantation, are also shown. Electrodes with impedance >2 MΩ or <10 kΩ were considered non-functional and were not included in these graphs. Impedances were inversely correlated with site size and median impedances of functioning electrodes remained stable within a range of 50–500 kΩ while implanted. Results of a Kruskal–Wallis test showed that site size was statistically significant (*p* < 0.01) in both groups, while the effect of time was not significant (*p* = 0.86 and 0.35 for groups 1 and 2, respectively.) **(B)** Percentage of implanted electrodes with impedances <2 MΩ and >10 kΩ. Each time point represents all implanted electrodes at each week, while electrodes from FMAs that suffered catastrophic failures were not included. Around 70–80% of group 1 and 90% of group 2 electrodes that remained implanted were functional at 12 weeks.

Figure 6A shows a summary of chronic impedance measurements of all electrodes in all animals over the lifetime of the implants. Out of 32 electrodes on each array, there were 8 electrodes of each site size. The line plot shows the median impedance of the electrodes of a particular site size while the error bars express the upper and lower quartiles of the data. As time progressed, the number of functioning electrodes decreased (Figure 6B). Neural recordings on electrodes with impedances >2 MΩ or <10 kΩ were extremely noisy and considered to be broken, and therefore were not included in chronic impedance data.

Electrical impedances, as expected, were inversely correlated with site size. The results of a Kruskal–Wallis ANOVA showed that site size had a significant effect on electrical impedance (*p* < 0.01 in both groups of electrodes). *Post hoc* testing using the Mann–Whitney *U* test confirmed that impedance measurements were larger for smaller site sizes, as shown in both groups. Impedances were not significantly related to post-implant time (*p* = 0.86 and 0.35 for groups 1 and 2, respectively), and overall, the impedances remained stable within a range of 50–500 kΩ while implanted.

As described earlier, increased electrical impedance was used to diagnose broken electrodes (impedances >2 MΩ or impedance <10 kΩ). Figure 6B shows the percentage of implanted electrodes that were considered functional as time progressed, separated by group and site size. Each time point represents all implanted electrodes at each week. While nearly 100% of electrodes were functional immediately after implant, this value decreased over time. Instances where complete failure of an array occurred due to broken lead wires were removed from this dataset. In group 1 electrodes, the percentage of functional electrodes slowly dropped to between 70 and 80% for all site sizes, while group 2 electrodes remained stable at 90% after 12 weeks. The large variation in the impedance measurements for some weeks may be attributed to a faulty connection within the adapter chain between the implanted electrode array and potentiostat, such as an electrical short (e.g., group 1, week 2) or broken lead (e.g., group 2, week 5).

## Discussion

The primary goal of this work was to assess the ability to record extracellular action potentials from populations of motor axons in the ventral roots using penetrating microelectrode arrays implanted chronically in lumbar spinal roots of adult cats. Because of notable differences in implant location, animal model, spike sorting techniques, and measurements of signal quality, it is difficult to compare this work with the performance of other types of implantable microelectrodes [e.g., Ref. (7, 18–21)]. Nonetheless, based on the results of this study, it is clear that it was possible to record single unit activity from populations of motor units in the ventral root for up to 12 weeks after implantation.

When successfully placed, many electrodes recorded activity from motor axons. However, accurate placement of electrodes in the ventral root was challenging to achieve, even using intraoperative recordings to guide placement. In post-operative testing, all of the implanted arrays contained at least one electrode that demonstrated activity during anesthetized recordings, suggesting that they were recording sensory activity from neurons in the dorsal root. Optimal placement of the arrays was difficult to achieve, but at least 20 motor units per array were recorded in 7 of 9 implanted animals. In addition, these well-targeted electrodes tended to be clustered together. Electrodes that recorded motor activity were statistically more likely to be neighbors of other electrodes that also recorded motor activity than those that did not.

The specifications for electrode design were also investigated to examine whether there was a relationship between an electrode’s site size and recording signal quality. It was originally expected that the larger electrodes would be more likely to record neural activity from multiple single units, since the probability of being near signal sources would be higher, albeit with lower SNR. On the other hand, it was expected that small electrodes would provide better unit isolation leading to high SNR recordings. The hypothesis was formulated upon the idea that larger site sizes would offer a higher likelihood of being positioned close to nodes of Ranvier where extracellular current densities are high, while smaller site sizes would have fewer nodes of Ranvier close to their electrode tip. However, in this study, there was no significant relationship between SNR and site size, as has been previously reported (17). In another study of the effects of site size on recording quality in the cortex (22), it was reported that the SNR increased with decreasing electrode tip length, but concluded that the optimum range for single unit recordings was 30–220 μm, without providing statistical comparisons of site sizes within that range. Finally, the single unit yields were also compared across all site sizes. Ultimately, it was found that site size was not a statistically significant factor for the number of units recorded and that all site sizes recorded units with similar SNR values.

While no statistically significant relationship between site size and SNR was found, there were reasons to expect that signal amplitudes and noise levels might vary with site size independently. Based solely on the higher impedances associated with smaller electrodes, it would be expected that smaller electrodes would have higher noise levels. Meanwhile, it was unclear whether the spatial averaging effects that should occur with larger electrodes along with their increased probability of being near a node of Ranvier would dominate the recorded signal amplitude. Interestingly, site size did not have a significant effect on noise levels in group 2 electrodes, nor did they influence signal amplitudes in either group. In group 2 electrodes, the noise levels did not follow impedance trends, suggesting that the source of the noise was predominantly background neuronal activity, not electrode thermal noise (23, 24). The somewhat larger site sizes associated with group 2 electrodes could have also contributed to these findings.

Electrical impedances were measured over time for all implanted electrodes to diagnose electrode integrity. Electrodes with impedances >2 MΩ or <10 kΩ were considered to be broken, as neural recordings on these channels were always extremely noisy, no single units neural activity was ever detected, and the impedance levels were significantly higher than the original manufactured specifications. Electrode impedance may increase for a number of biotic [e.g., encapsulation (25–27), immune response to foreign body (27, 28)] and abiotic [e.g., broken electrode tips (29), insulation damage (30), breaks in wire bundle] failure modes. It was hypothesized that smaller site sizes would be more quickly affected by encapsulation, and therefore, would demonstrate earlier biotic failure than larger recording sites. Since the mechanical structure of all electrodes and leads were similar, the chance of abiotic failure modes was hypothesized to be the same for all implanted electrodes.

For all site sizes, the electrical impedances remained stable during the post-implantation period, with the smaller sites have a higher value, similar to previous studies that examined the relationship between recording site size and impedance (17). This was consistent for all cats and implanted arrays. Statistical analysis showed that site size was a statistically significant factor (*p* < 0.01), while post-implant time was not. While microstimulation studies have revealed a pattern of a sharp increase in impedance within the first 2–3 weeks post-implantation followed by a decay to some baseline (18, 21, 31, 32), the findings here follow impedance patterns shown in long-term implantation studies applying microelectrode arrays solely for neural recording (17, 20).

This study tracked the percentage of functional electrodes over time based on electrical impedances and found that the rates of failure for all site sizes were not statistically different. While electrodes did break in many animals and only two cats completed the planned 12-week implant time, the percentage of implanted electrodes that remained functional stayed relatively stable, suggesting that solving technical issues related to lead failure could allow a large number of neural recordings. In the two animals that completed the planned 12-week implants, 80% of the implanted electrode remained viable.

This study demonstrated the feasibility of achieving chronic recordings from motor axons in the ventral root. However, some limitations still remain. Implanting FMAs through intact epineurium proved to be difficult and required high-speed insertion methods. In part, because of this difficulty, consistent electrode targeting has remained a challenge. Observations of perfused spinal nerve tissue have shown that electrodes can pass through the ventral surface of the ventral root and that these electrode shafts can bend, presumably as a result of mechanical contact with the spinal canal. These results suggest that other approaches to implantation or other approaches of implantation, such as targeting the ventral root from the lateral or ventral side, may be more appropriate for accessing the ventral root.

Despite these limitations, this study presents the first chronic neural recordings from multi-channel microelectrode arrays in the ventral roots. These results show that it is possible to record from motor axons in the ventral root, and that the neural signals can be detected by variety of electrode site sizes. These findings suggest that the ventral root is a potentially feasible site for a peripheral motor interface.

Further work with these devices will focus on improving the approach to the ventral root such that proper and accurate targeting can be achieved more consistently. Additionally, a detailed analysis of histological data will be performed to identify any biotic causes to changes in the unit yield observed over the course of the study. Future studies will focus on the development of methods to decode the motor commands associated with ventral root recordings in order to attain useful control signals for devices such as prosthetic limbs and orthoses.

## Conflict of Interest Statement

The authors declare that the research was conducted in the absence of any commercial or financial relationships that could be construed as a potential conflict of interest.
